# Assessment of Autism Zebrafish Mutant Models Using a High-Throughput Larval Phenotyping Platform

**DOI:** 10.3389/fcell.2020.586296

**Published:** 2020-11-23

**Authors:** Alexandra Colón-Rodríguez, José M. Uribe-Salazar, KaeChandra B. Weyenberg, Aditya Sriram, Alejandra Quezada, Gulhan Kaya, Emily Jao, Brittany Radke, Pamela J. Lein, Megan Y. Dennis

**Affiliations:** ^1^Genome Center, Department of Biochemistry and Molecular Medicine, School of Medicine, University of California, Davis, Davis, CA, United States; ^2^Integrative Genetics and Genomics Graduate Group, University of California, Davis, Davis, CA, United States; ^3^Sacramento State RISE Program, California State University, Sacramento, Sacramento, CA, United States; ^4^Department of Molecular Biosciences, School of Veterinary Medicine, University of California, Davis, Davis, CA, United States; ^5^MIND Institute, School of Medicine, University of California, Davis, Davis, CA, United States

**Keywords:** *Danio rerio*, phenotyping, CRISPR, developmental disorders, seizure, VAST, DanioVision, zebrafish

## Abstract

In recent years, zebrafish have become commonly used as a model for studying human traits and disorders. Their small size, high fecundity, and rapid development allow for more high-throughput experiments compared to other vertebrate models. Given that zebrafish share >70% gene homologs with humans and their genomes can be readily edited using highly efficient CRISPR methods, we are now able to rapidly generate mutations impacting practically any gene of interest. Unfortunately, our ability to phenotype mutant larvae has not kept pace. To address this challenge, we have developed a protocol that obtains multiple phenotypic measurements from individual zebrafish larvae in an automated and parallel fashion, including morphological features (i.e., body length, eye area, and head size) and movement/behavior. By assaying wild-type zebrafish in a variety of conditions, we determined optimal parameters that avoid significant developmental defects or physical damage; these include morphological imaging of larvae at two time points [3 days post fertilization (dpf) and 5 dpf] coupled with motion tracking of behavior at 5 dpf. As a proof-of-principle, we tested our approach on two novel CRISPR-generated mutant zebrafish lines carrying predicted null-alleles of *syngap1b* and *slc7a5*, orthologs to two human genes implicated in autism-spectrum disorder, intellectual disability, and epilepsy. Using our optimized high-throughput phenotyping protocol, we recapitulated previously published results from mouse and zebrafish models of these candidate genes. In summary, we describe a rapid parallel pipeline to characterize morphological and behavioral features of individual larvae in a robust and consistent fashion, thereby improving our ability to better identify genes important in human traits and disorders.

## Introduction

Zebrafish (*Danio rerio*) are freshwater teleost fish widely used to study genes important in human diseases and traits, including neurological disorders such as autism-spectrum disorder [ASD; see review from Meshalkina et al. (2018)], intellectual disability (ID), and epilepsy [see review from Grone and Baraban (2015); Sakai et al. (2018)]. The zebrafish represent a robust model for disease studies with significant genetic homology (∼70–80% of orthologous genes) with humans (Howe et al., 2013; Flicek et al., 2014). Furthermore, their small size, robust reproduction, and transparent appearance during embryonic development (Kimmel et al., 1995) facilitates more rapid and higher-throughput experiments compared to other commonly used model organisms such as rodents. The highly efficient nature of CRISPR editing applied to zebrafish (Chang et al., 2013; Jao et al., 2013; Peng et al., 2014) has led to an exponential increase in the numbers of genes that can be functionally tested via direct perturbation in embryos followed by phenotypic scoring. Studies can be performed in G_0_ mosaic fish due to the efficient bi-allelic disruption and phenotype presentation (Jao et al., 2013; Shah et al., 2015; Wu et al., 2018). Due to the high level of mosaicism, stable lines are readily generated allowing direct genotype-to-phenotype comparisons (Varshney et al., 2015). These approaches have successfully been applied to multiple genes in parallel in order to study their impact on neurodevelopmental features in zebrafish (Shah et al., 2015; Thyme et al., 2019). The most recent example utilized CRISPR to characterize 132 human schizophrenia gene orthologs in stable mutant zebrafish lines followed by assessment of brain morphometry, neuronal activity, and behavior, demonstrating the potential for large-scale studies of gene functions in morphology and behavior (Thyme et al., 2019). Although significant, this landmark study required considerable resources to house, cross, and phenotype individual stable mutant lines on such a large scale.

With the ease of CRISPR gene editing, our main bottleneck in analyses is quick, quantitative, and high-throughput phenotyping approaches. Currently, there are few automated imaging systems for zebrafish larvae. The Vertebrate Automated Screen Technology BioImager platform (VAST BioImager^TM^) uses a capillary-based flow system to load and image 2–7 days post fertilization (dpf) zebrafish larvae coupled with 360^° rotation (Pardo-Martin et al., 2010; Teixidó et al., 2019). Characterization of the VAST system has demonstrated that larvae are not affected physically or developmentally by passage through the system tubing during imaging (Pulak, 2016). Furthermore, a number of systems have utilized successful motion monitoring of behavior of up to 96 larvae in parallel housed in plate wells, including DanioVision video tracking coupled with EthoVision software (Noldus et al., 2001). Although VAST and DanioVision provide consistent results (Teixidó et al., 2019), previous literature has not evaluated the combined use of VAST and a behavioral-tracking system to phenotype zebrafish mutants.

Here, we developed a high-throughput phenotyping protocol to maximize the use of multiple quantitative measurements of the same zebrafish at two developmental time points using automated imaging (VAST) and behavioral assays (DanioVision) to test for gross morphological defects and seizure susceptibility, respectively. To optimize our approach, we performed baseline experiments on a well characterized wild-type (WT) zebrafish line, NHGRI-1 (LaFave et al., 2014), and subsequently tested our strategy using a novel zebrafish null model of ASD-candidate gene *SYNGAP1B*. Morpholino knockdown of the zebrafish ortholog *syngap1b* was previously shown to lead to sporadic seizures, reduced brain ventricle size, and microcephaly (Kozol et al., 2015). Mutations in orthologs of *syngap1b* in humans and rodents lead to ASD morphological phenotypes, including seizures and microcephaly (Hamdan et al., 2011; Kozol et al., 2015). We also tested our system using a novel zebrafish null mutant model of *SLC7A5*, a gene implicated in ASD, microcephaly, and epilepsy in humans with neurodevelopmental phenotypes also observed in mice (Tǎrlungeanu et al., 2016). Overall, we found that our combined phenotyping platform was capable of detecting enhanced drug-induced seizure behavior in both mutant models in addition to developmental abnormalities. Coupled with CRISPR gene editing, higher-throughput screening approaches will be instrumental in scaling up functional assessment of genes important in neurodevelopment using zebrafish.

## Materials and Methods

### Zebrafish Care and Husbandry

All experimental procedures and animal handling were performed in accordance with the University of California Davis and AAALAC guidelines and were approved by the Institutional Animal Care and Use Committee. Adult zebrafish from an NHGRI-1 background (LaFave et al., 2014) were housed in a modular zebrafish system (Aquaneering, San Diego, CA, United States), and all fish were kept in a 10-h dark/14-h light cycle, and 28 ± 0.5°C filtered, UV sterilized water (Kimmel et al., 1995). NHGRI-1 is an isogenic line that has been fully sequenced and was used for all of our studies. Adult zebrafish were monitored and fed two times a day: flakes (Zebrafish select diet, Aquaneering, San Diego, CA, United States) and brine shrimp (Aquaneering, San Diego, CA, United States). For experimentation, we collected eggs from randomly assigned NHGRI-1 pairs from at least 10 breeding pairs and a total of seven experiments for WT. For *syngap1b* mutants, we collected eggs from four breeding pairs over a total of two experiments. For *slc7a5* stable mutants, we collected eggs from five breeding pairs over a total of three experiments. For the G_0_ *slc7a5* mutants, we collected eggs from seven WT breeding pairs from one experiment and performed injections. For breeding, we kept our adult female and male fish separate overnight in a 1-L crossing tank (Aquaneering, item #ZHCT100, San Diego, CA, United States) and released them by removing the divider in the crossing tank when the lights turned on the next morning. We collected the fertilized embryos within 1 h after the fish were released. Eggs were then rinsed two times with embryo water (0.03% Instant Ocean salt in deionized water at 28 ± 0.5°C) to remove debris and plated at a maximum of 100 embryos per plate (Wilson, 2012) in standard (100 × 15 mm) Petri dishes in embryo water and incubated at 28 ± 0.5°C until they were used for experiments at 3 and 5 dpf. We used a Leica dissecting microscope (Buffalo Grove, IL, United States) to quantify, stage, monitor, and remove dead embryos daily, following previously described methods (Kimmel et al., 1995). Embryos were mixed from all clutches obtained (from crossed pairs) and randomly assigned to experimental groups and/or CRISPR microinjections.

### Chemicals

Tricaine methanesulfonate (MS-222, purity ≥99%) (Thermo Fisher Scientific, Waltham, MA, United States) was prepared as 2x stocks in embryo water and diluted to its final concentration (0.02 mg/ml for VAST or 0.125% for adult zebrafish genotyping) on the day of the experiments. All zebrafish larvae that were used for morphometry experiments were exposed to MS-222 through the VAST (Union Biometrica, Holliston, MA, United States) platform. Pentylenetetrazol (PTZ; purity ≥99%, Sigma-Aldrich) and bicuculline (BCC; purity ≥97%, Sigma-Aldrich) solutions were prepared on the day of the experiments in embryo water, and their concentrations were selected based on previous studies (Baraban et al., 2005; Cassar et al., 2017) and our baseline exposure experiments.

### VAST

For morphological measurements, we used the VAST instrument equipped with a large particle sampler (LP sampler^TM^) on fish at 3 or 5 dpf. We collected images using the VAST built-in camera and measured eye area (EA), head width, and body length (L) from single fish that were imaged ventrally and dorsally. Our parameters for VAST imaging at 3 and 5 dpf were similar with the exception of the template images, which correspond to the correct age and L, which was 3.8 mm for 3 dpf fish and 4 mm for 5 dpf larvae, following a previously described protocol (Pulak, 2016). Images were processed with FishInspector imaging software [version 1.03 (Teixidó et al., 2019)] to extract all established shapes for each measured larvae. After this, specific morphometric measurements were extracted using the R package Momocs (Bonhomme et al., 2014) with the known capillary width as a scale. Some images in FishInspector required manual correction performed by using the “edit” function. EA represented the average of both eyes, extracted by tracing the outside of each eye and measuring the area of this shape. L and head width were measured using the outline of the fish in the dorsal view and extracting the distance between the farthest most left and right (L) and top and bottom (head width) points of the fish, respectively. Pericardium area was measured by tracing the cardial sac and extracting its area. Yolk area was measured by tracing the yolk, which presents as yellow in color, and extracting its area. Manual measurements were also performed for optic tectum (OT; the distance directly behind the eyes) and telencephalon (Tel; the distance directly between the eyes) following a previously described method (Näslund, 2014). FIJI was used for the manual measurements using a set scale, taking the width of the capillary (0.6 mm) as a reference.

### Behavioral Assay

Locomotor behavior screening of zebrafish larvae in round 96-well plates was performed in WT NHGRI-1 and stable mutant lines at 5 dpf using the DanioVision (Noldus, Leesburg, VA, United States) instrument. Zebrafish larvae were individually placed (one fish per well) in 100 μl of embryo water. Experiments were conducted for a total of 1 h. Habituation was performed for 10 min by incubating the 96-well plate with larvae in the DanioVision chamber (28.5°C) prior to every experiment. Then 100 μl of embryo water (0 mM PTZ) or 100 μl of 4, 20, or 30 mM PTZ (final concentration of 2, 10, or 15 mM) was added for a final volume of 200 μl per well. For experiments with BCC, addition of 100 μl of embryo water (0 μM BCC) or 100 μl of 10, or 20 μM BCC (final concentration of 5 and 10 μM) was added for a final volume of 200 μl per well. The experiment started immediately following exposure in the temperature controlled (28.5°C) DanioVision chamber. After 45 min of motion tracking with light, we implemented light flashes (three flashes for 10 s every 10 s) to assay light-induced seizurogenic activity (Zheng et al., 2018). Locomotor behavioral activity, quantified as total distance moved per min over 1 h, was recorded with EthoVision 10.0 tracking software.

### Zebrafish CRISPR Mutant Generation

Mutant zebrafish lines (*syngap1b* and *slc7a5*) were created by microinjections of ribonucleic proteins (RNPs) composing of Cas9 enzyme coupled with single guide RNAs (gRNAs) (Integrated DNA Technologies, Coralville, IA, United States) following the manufacturer’s protocol (Integrated DNA Technologies; see Supplementary Table S1 for description of gRNAs). Microinjections were performed at the one-cell stage as previously described (Jao et al., 2013) using needles from a micropipette puller (Model P-97, Sutter Instruments, Novato, CA, United States), and an air injector (Pneumatic MPPI-2 Pressure Injector, Eugene, OR). Injection mixes contained 1.30 μl of Cas9 enzyme (20 μM, New England BioLabs), 1.60 of prepared gRNAs, 2.5 μl of 4x Injection Buffer (0.2% phenol red, 800 mM KCl, 4 mM MgCl_2_, 4 mM TCEP, 120 mM HEPES, pH 7.0), and 4.6 μl of nuclease-free water. In the *slc7a5* “pooled” experiment, we injected embryos from the same crosses with three gRNAs (1.60 μl for each) and compared to a “mock” injected with the same mix sans gRNAs.

### DNA Isolation and Genotyping

In order to determine the exact genetic alteration in our mutants, we performed Illumina amplicon sequencing through the Massachusetts General Hospital DNA Core Facility (Cambridge, MA, United States) using the primers found in Supplementary Table S1. Briefly, a ∼200-bp region surrounding the gRNA target site was amplified, purified using magnetic beads (AMPure XP, Beckman Coulter, Indianapolis, IN, United States), and Illumina sequenced with 2 × 150 bp reads. Paired reads were aligned to the reference target region using *bwa* (Li and Durbin, 2009) and the zebrafish reference genome (GRCz11/danRer11). Specific alleles were determined using the R package CrisprVariants (Lindsay et al., 2016). Genotyping stable lines was performed via PCR and SDS polyacrylamide gels or high-resolution melt curves (primers listed in Supplementary Table S1). Adult zebrafish were anesthetized in 0.125% MS-222 and a small piece of caudal fin tissue (<50% of caudal fin) was dissected for crude DNA isolation. Briefly, 100 μl of 50 M NaOH was added to the fin clip and incubated in the solution at 95°C for 20 min followed by a 10 min 4°C incubation. Then 10 μl of 5 M HCl was added to the sample to neutralize the solution. The samples were vortexed for 5 s, and the isolated DNA was used for PCR amplification and other downstream procedures. PCR amplification was performed using DreamTaq Green PCR master mix following the manufacturer’s protocol (Thermo Fisher Scientific, Waltham, MA, United States) using the Bio-Rad T100^TM^ thermal cycler. High-resolution melt analysis was performed using SYBR green (Thermo Fisher Scientific) using the Bio-Rad CFX96 Touch^TM^ Real Time PCR System.

### Identification of Potential CRISPR Off-Targets

CIRCLE-seq libraries for each gRNA and a control (Cas9 enzyme only) were prepared following the described protocol (Tsai et al., 2017; Lazzarotto et al., 2018). Illumina sequencing (Novogene, Sacramento, CA, United States) of the libraries generated ∼5 million reads per sample that were mapped to the zebrafish reference genome (GRCz11/danRer11) to define predicted off-target sites relative to the control sample following the established CIRCLE-Seq bioinformatic pipeline (data has been deposited to the European Nucleotide Archive Accession PRJEB40101). Once potential off-target sites were defined, we PCR amplified a ∼500-bp region of G_0_ mosaic mutants of the top sites inside genes predicted by CIRCLE-seq (13 for *syngap1b* and 6 for *slc7a5*) (primers listed in Supplementary Table S1) and ran a polyacrylamide gel electrophoresis to identify the presence of indels at these sites by the formation of heteroduplexes (Zhu et al., 2014). Additionally, if heteroduplexes were detected, PCR reactions were cleaned-up using Ampure XP magnetic beads (Beckman Coulter), and Sanger sequenced (Genewiz, San Diego, CA, United States). We did not observe off-target indels in these sites when compared to WT siblings.

### Molecular Characterization of Mutants

To quantify abundance of RNA of targeted genes in stable mutant lines, 5-dpf larvae from a cross of heterozygous mutants were quickly euthanized in a Petri dish on dry ice, and a small piece of caudal fin tissue was carefully removed from each larva for DNA extraction and genotyping via high-resolution melt curves and SDS polyacrylamide gels, as previously described (primers listed in Supplementary Table S1). The larvae were individually placed in RNAlater (Thermo Fisher Scientific, Waltham, MA, United States), and RNA extraction was performed using the RNAeasy kit (Qiagen) with gDNA eliminator columns for DNA removal. RNA concentration from all samples was obtained with the Qubit RNA BR kit (Thermo Fisher Scientific, Waltham, MA, United States). RNA levels of *syngap1b* or *slc7a5* in stable mutant lines were evaluated using a quantitative PCR with the Luna Universal One-Step RT-qPCR kit (New England BioLabs) following the manufacturer’s protocol. Fold change values were obtained using the ΔΔCq method, with raw Cq values normalized with expression of housekeeping gene β-actin (primers listed in Supplementary Table S1) and represented as fold changes to the average of the WT samples.

### Statistical Analysis

Statistical analyses were performed using R (3.5.0) for morphometric measurements and GraphPad Prism 8 software (GraphPad software, La Jolla, CA, United States) for all behavioral assays. Pearson correlation tests were used to compare manual and automated measurements. The effect of factors on morphometric measurements was evaluated using multiple regression tests with the measurement as the response variable. Principal component analysis (PCA) was performed using the R library PCAmixdata (Chavent et al., 2014), which integrates PCA with multiple correspondence analyses (MCA) to evaluate quantitative (EA, L, Tel, and OT) and qualitative (genotype) data simultaneously. Analysis of variance (ANOVA) was utilized to extract the effect of genotype in morphometric or behavioral measurements, followed by Tukey’s *post hoc* tests to identify differences between genotypic groups. All models tested in this project controlled for inter-batch differences by adding “Experiment” as a covariate. Kruskal–Wallis was used for behavioral analysis of mutants with a Dunn’s multiple comparison *post hoc* test performed for comparisons between genotypes of stable mutants. Mann–Whitney tests were performed for comparisons of behaviors in the pilot experiment of PTZ and BCC as well as for mosaic G_0_ pool versus mock-injected larvae. Significance from tests was defined by an alpha of 0.05.

## Results

### High-Throughput Phenotyping of WT Zebrafish Larvae

#### Morphometric Phenotypes

**FIGURE 1:**
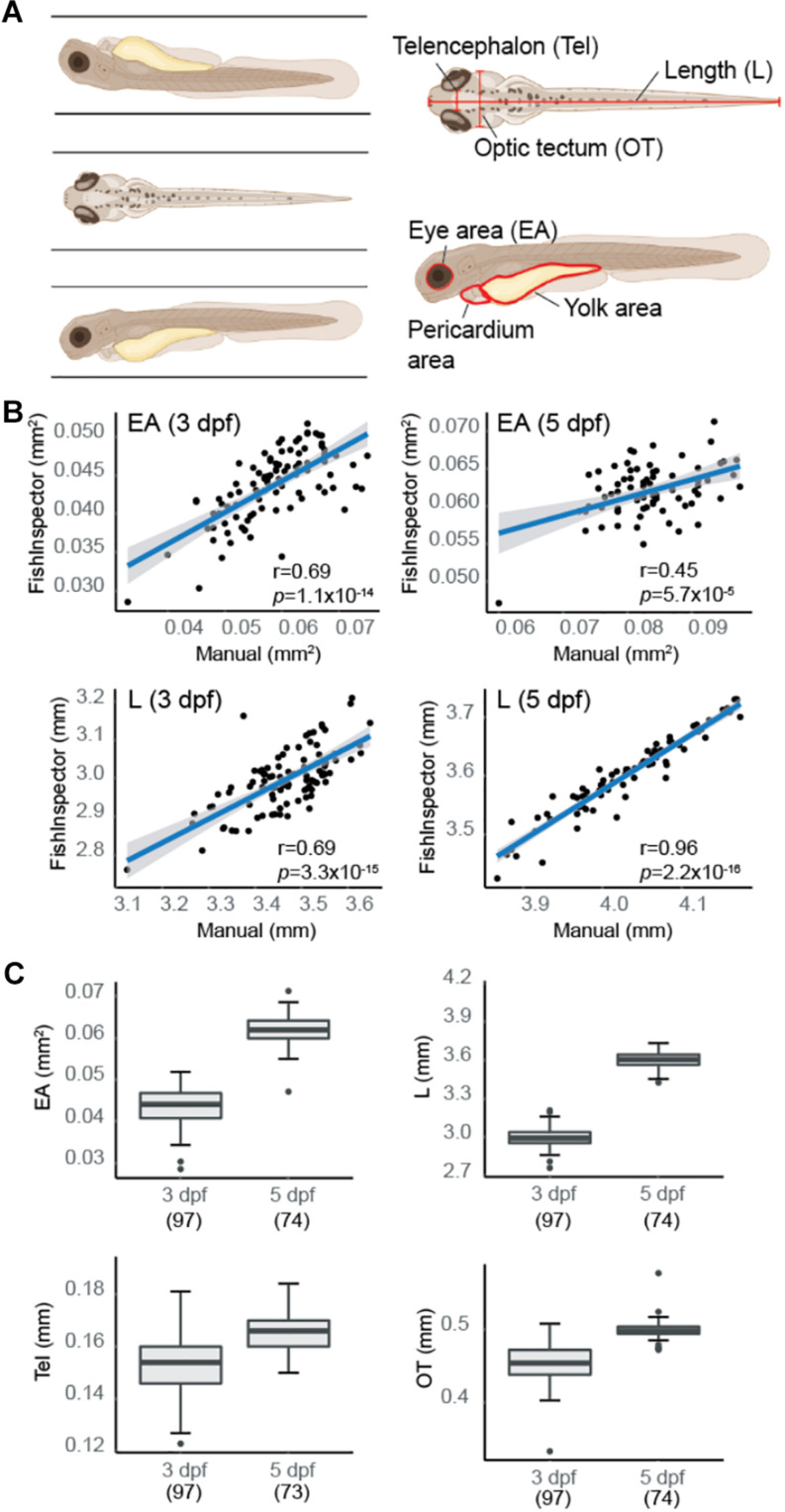
Morphometric-phenotype assessment of larvae at 3 days post fertilization (dpf) and 5 dpf. **(A)** Larval fish were imaged in three orientations (dorsal, right lateral, and left lateral) using the VAST system. Morphometric features were obtained manually using Fiji [body length (L), eye area (EA), telencephalon (Tel), and optic tectum (OT)] and automatically using FishInspector (L, EA, head width, pericardium area, and yolk area). **(B)** Manual and automated measurements were compared with L and EA (averaged between both eyes) showing high correlations at 3 dpf (*n* = 97) and 5 dpf (*n* = 73) (Pearson correlation). Blue line represents the fit of a linear model with 95% confidence intervals. **(C)** Boxplots pictured represent distribution of measurements for larval morphometric features obtained via automated (L and EA) and manual (Tel and OT) methods. Boxplots include the median value (dark line), and the whiskers represent a 1.5 interquartile range.

To optimize anatomical quantifications of WT (NHGRI-1) zebrafish larvae at 3 dpf (*n* = 100) and 5 dpf (*n* = 78) using the VAST imaging platform, we performed manual measurements of eye area (EA), length (L), as well the width of the head at two sites previously shown to correlate with the telencephalon (Tel) and optic tectum (OT) (Näslund, 2014; Figure 1A). Previous work has demonstrated that larval development is not affected by passage through the VAST system tubing (Pulak, 2016). Using the same images, we also extracted measurements using automated analysis software FishInspector (Teixidó et al., 2019) for EA, L, and head width (defined as the widest part of the fish; see section “Materials and Methods”), as well as two additional features, pericardium area, and yolk area (Supplementary Figure S1A). By examining the normal distribution of data for a subset of features (EA and L), we flagged a proportion of larvae as outliers (3 dpf: 10%, 5 dpf: 7.7%; Supplementary Figure S1B). By visual inspection of all images, we found that less than half of the outliers represented technical errors in imaging using the VAST (e.g., full length of fish not included in the image; 3 dpf: 3%, 5 dpf: 4%), while the remaining outliers represented FishInspector software errors in automatically defining morphological features. Taking a closer look at additional measurements, including yolk and pericardium areas, as well as head width, we found that >10% of values also fell outside of the confidence interval based on a normal distribution, with the majority due to inaccuracies in FishInspector feature tracing. As such, we manually corrected FishInspector traces for EA, L, and head width, but chose not to move forward with yolk and pericardium area measurements due to difficulties in manual manipulations of these features in the software. Using this curated dataset, we showed significant correlations between our original manual measurements of EA [3 dpf: *r*_(__94__)_ = 0.69, *p* = 1.1 × 10^–14^; 5 dpf: *r*_(__72__)_ = 0.45, *p* = 5.7 × 10^–5^] and L [3 dpf: *r*_(__95__)_ = 0.69, *p* = 3.3 × 10^–15^; 5 dpf: *r*_(__72__)_ = 0.96, *p* < 2.2 × 10^–16^] with our corrected automated measurements (Figure 1B). Additionally, we found that our manual measures of Tel correlated better to automated measures of head width at 5 dpf [3 dpf: *r*_(__95__)_ = 0.13, *p* = 0.196; 5 dpf: *r*_(__71__)_ = 0.30, *p* = 0.0087], while OT correlated with head width at both ages [3 dpf: *r*_(__95__)_ = 0.46, *p* = 1.6 × 10^–6^; 5 dpf: *r*_(__72__)_ = 0.35, *p* = 0.0024]. Based on these results, we proceeded with quantifying VAST images via FishInspector automated measurements for EA and L and manual measurements of Tel and OT as a proxy for brain size (Figure 1C).

#### Behavioral Phenotypes

**FIGURE 2:**
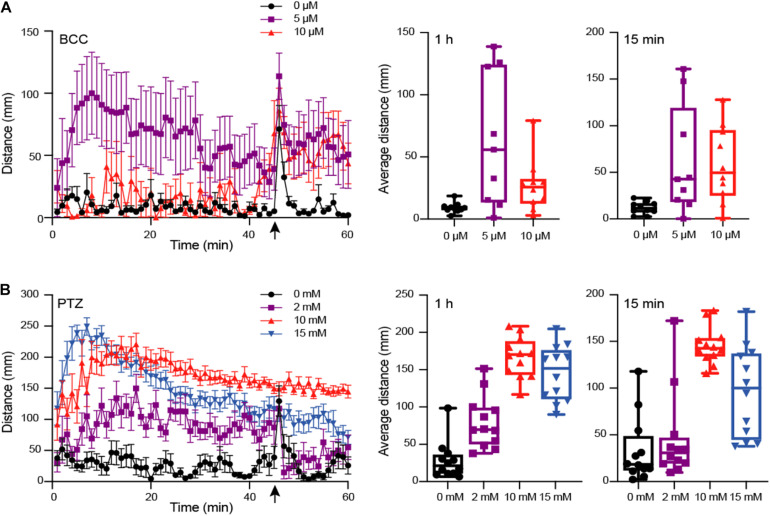
Behavioral phenotype assessment of larvae at 5 dpf using motion tracking. Larvae treated with varied concentrations of GABA_*A*_ antagonizing drugs **(A)** Bicuculline (BCC) (0 to 10 μM; *n* = 10) or **(B)** Pentylenetetrazol (PTZ) (0 to 15 mM; *n* = 12), respectively, were tracked using DanioVision and total distance moved per minute measured for 1 h. With 15 min remaining (indicated by an arrow), larvae were subjected to flashing lights for 1 min. The average movement per fish across 60 min and in the last 15 min following flashing lights are indicated as boxplots for each drug concentration. Left plots include the mean distance per minute and with error bars representing standard error of the mean.

A number of studies have used motion tracking to characterize locomotor behavior and seizure susceptibility in order to assay neurological phenotypes in zebrafish larvae (Afrikanova et al., 2013; Kozol et al., 2015; Zheng et al., 2018). Previous work using the chemical convulsant PTZ has identified a concentration-dependent difference in distance, velocity, and swim activity (Baraban et al., 2005; Bandara et al., 2020). To verify our ability to recapitulate these results, we subjected larvae (5 dpf) to varying concentrations of two GABA_*A*_ receptor antagonists, PTZ (2, 10, and 15 mM; *n* = 12), a selective antagonist, as well as BCC (0, 5, and 10 μM; *n* = 10), a non-selective antagonist. We then tracked their movement over 1 h with a brief flashing of lights after 45 min using the DanioVision motion-tracking system (Figure 2). We quantified the average distance of larval movement per min over the entire 1 h and over the last 15 min after being subjected to flashing light, respectively. For maximum concentrations of BCC (10 μM) and PTZ (15 mM), we observed significantly reduced movement overall versus lower concentrations in the final 15 min (*p* < 0.05 for BCC and PTZ; Mann–Whitney test) and over the entire 1 h (*p* < 0.05 for BCC only; Mann–Whitney test), suggesting that the larvae experienced what is known as stage III clonic-like seizures, consistent with published results with 15 mM PTZ (Baraban et al., 2005). We note that in our experiment, the larvae treated with 15 mM PTZ initially experienced greater movement that gradually subsided at ∼10 min, consistent with our finding that significant differences were only observed with this treatment across the final 15-min comparison. We found the strongest effect on distance moved (mean distance = 166.88 mm) with the lowest variance (SD = 27.17) using 10 mM PTZ, a concentration with little morbidity, in concordance with previous studies (Baraban et al., 2005; Afrikanova et al., 2013).

### Optimization of a Combined Morphological and Behavioral Phenotyping Platform

**FIGURE 3:**
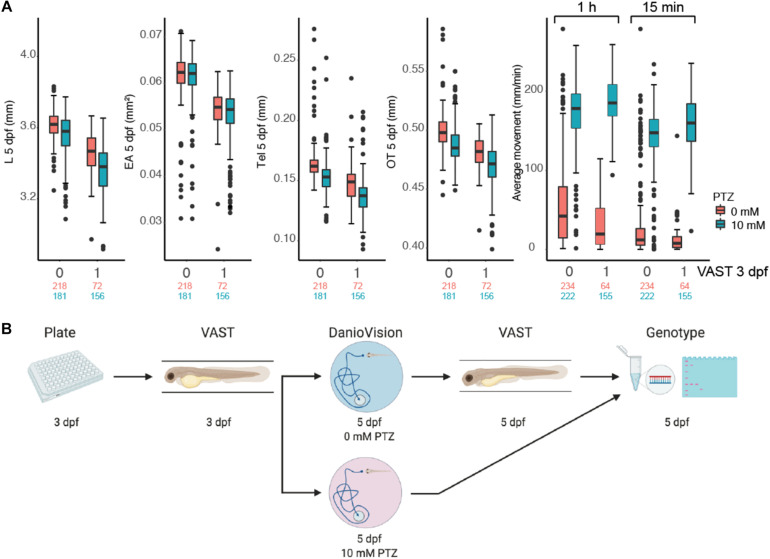
High-throughput zebrafish larvae phenotyping platform. **(A)** Impact of morphometric (L, EA, Tel, OT) and behavioral (average movement for the entire 1 h and the final 15 min following flashing lights) measurements for 5 dpf larvae for fish not subjected (0) and subjected (1) to VAST measurements at 3 dpf are shown as boxplots. Behavior was also plotted at varied PTZ concentrations (red = 0 mM and blue = 10 mM). Total numbers of measured larvae are indicated beneath plots subjected to 0 mM (red) or 10 mM (blue) PTZ treatment. Boxplots for morphological and behavioral measurements include the median value (dark line), and the whiskers represent a 1.5 interquartile range. **(B)** The final combinatorial phenotyping platform is pictured, which combines two morphometric measurements (3 and 5 dpf) and behavioral tracking (5 dpf) with 0 mM and 10 mM PTZ, followed by genotyping of larvae.

Although previous studies have shown the utility of VAST and DanioVision platforms to independently characterize phenotypes in a high-throughput fashion, we sought to identify optimal parameters to utilize these approaches in combination at multiple developmental time points in individual zebrafish larvae while also minimizing impacts on traits. WT NHGRI-1 embryos (*n* = 992) collected across seven experiments were placed in 96-well plates and subjected to varied measurements/treatments, including VAST imaging at 3 dpf, behavioral tracking at 5 dpf using the DanioVision with (10 mM) or without (0 mM) PTZ, and VAST imaging at 5 dpf (Figures 3A, Supplementary Figure S2, and Supplementary Table S2). Using our combined dataset, we found that larvae subjected to VAST at 3 dpf exhibited smaller measurements at 5 dpf for EA (15.2% decrease, *p* = 4.85 × 10^–4^), Tel (13.6% decrease, *p* = 1.36 × 10^–2^), and OT (4.5% decrease, *p* = 7.77 × 10^–4^), in addition to a notable decrease in the average movement observed during the behavioral assays (0 mM PTZ; average movement 1 h: 47.7% decrease, *p* = 0.046, average movement last 15 min: 60.9% decrease, *p* = 0.017) (Table 1, Figures 2A, and Supplementary Figure S3). Treating larvae with PTZ during our behavioral screen also had an effect on morphometric measurements at 5 dpf, with larvae exhibiting a reduction of 4.3% in EA (*p* = 8.77 × 10^–3^), 3.3% in L (*p* = 3.89 × 10^–9^), 10.2% in Tel (*p* = 2.28 × 10^–3^), and 3.1% in OT (*p* = 5.53 × 10^–4^). Additionally, we observed an increase in mortality (∼4%) for PTZ-treated versus untreated larvae. As such, we moved forward with a final phenotyping platform that included VAST imaging at 3 dpf followed by motion tracking at 5 dpf with half of the larvae treated with (10 mM) or without (0 mM) PTZ. To minimize PTZ effects on morphometric measurements and mortality, we performed final VAST imaging at 5 dpf only in larvae not treated with the drug (Figure 3B). We determined that ∼50 fish are needed per genotype in order to detect a >4% change between groups for morphometric features at 5 dpf at 80% power using our “optimized” assay, though for certain traits (OT and Tel), far fewer fish are required (Supplementary Table S3 and Supplementary Figure S4).

**TABLE 1:** Summary effect size and significance for each treatment per measurement.

**Morphometric measurement (5 dpf)**	Complete model*			**VAST at 3 dpf**		**Behavior**		**PTZ**	
	***F* (df)**	***p*-value**	***R*^2^**	**Beta**	***p*-value**	**Beta**	***p*-value**	**Beta**	***p*-value**
Average EA (mm^2^)	43.98 (9, 617)	*p* < 2.2 × 10^–16^	0.382	−0.00553	**4.85 × 10^–4^**	0.0006	0.477	−0.0020	**8.77 × 10^–3^**
				Yes = 228, No = 399		Yes = 566, No = 61		10 mM = 337, 0 mM = 290	
L (mm)	69.98 (9, 617)	*p* < 2.2 × 10^–16^	0.498	−0.06075	0.0525	0.0316	0.068	−0.0923	**3.89 × 10^–9^**
				Yes = 228, No = 399		Yes = 566, No = 61		10 mM = 337, 0 mM = 290	
Tel (mm)	35.71 (9, 617)	*p* < 2.2 × 10^–16^	0.333	−0.01152	**0.0136**	−0.005386	**0.037**	−0.0080	**5.53 × 10^–4^**
				Yes = 228, No = 399		Yes = 566, No = 61		10 mM = 337, 0 mM = 290	
OT (mm)	39.84 (9, 615)	*p* < 2.2 × 10^–16^	0.359	−0.01583	**7.77 × 10^–4^**	−0.0004	0.866	−0.0071	**2.28 × 10^–3^**
				Yes = 226, No = 399		Yes = 564, No = 61		10 mM = 335, 0 mM = 290	
1 h average distance (mm)	186.5 (8, 666)	*p* < 2.2 × 10^–16^	0.688	9.916	**0.046**	–	–	119.6	**<2 × 10^–16^**
				Yes = 219, No = 456		–		10 mM = 377, 0 mM = 298	
15 min average distance (mm)	184.3 (8, 666)	*p* < 2.2 × 10^–16^	0.685	11.712	**0.017**	–	–	122.7	**<2 × 10^–16^**
				Yes = 219, No = 456		–		10 mM = 377, 0 mM = 298	

*^∗^The “complete model” also included “Experiment” as a variable to reduce inter-batch effects (see section “Materials and Methods”). Values in bold represent *p* < 0.05.*

### Proof of Concept Using a Stable Zebrafish Mutant Model of *SYNGAP1*

**FIGURE 4:**
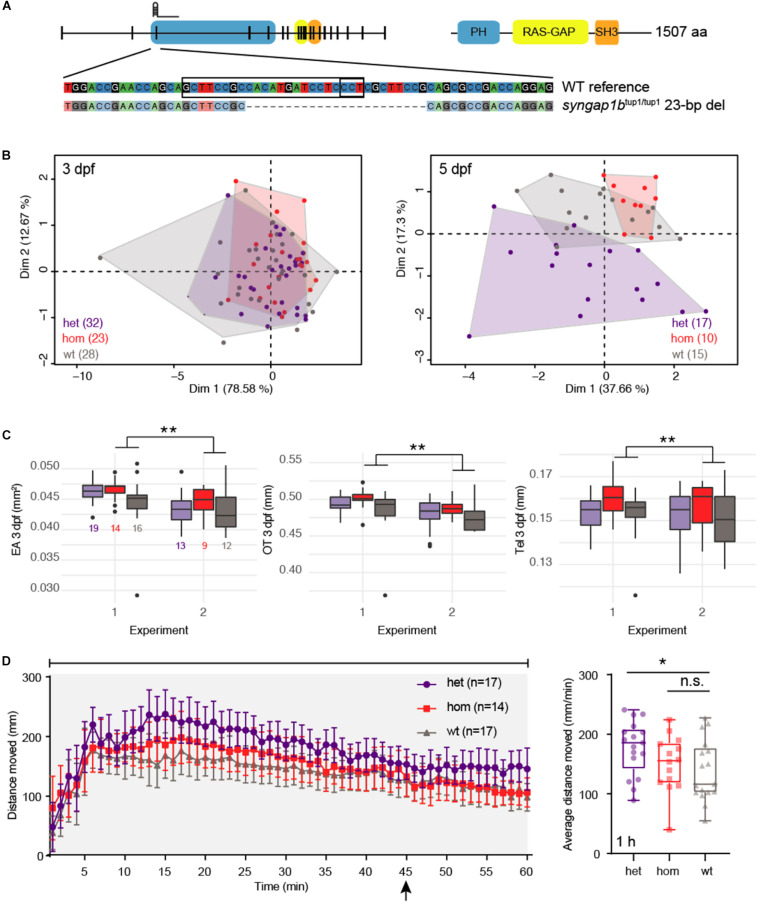
*syngap1b* CRISPR mutants exhibit developmental defects and enhanced seizures. **(A)** The zebrafish mutant line *syngap1b*^tup1/tup1^ was generated using a single gRNA targeting the third exon of the gene resulting in a 23-bp deletion leading to a frameshift. **(B)** Principal component analysis (PCA) of combined morphometric traits at 3 and 5 dpf colored by genotype. **(C)** The three traits found to be significantly increased in het (purple) and hom (red) mutant versus WT (gray) siblings included EA, OT, and Tel at 3 dpf, displayed as box plots with total numbers of larvae measured indicated in the EA plot (^∗∗^*p* < 0.01 using a Tukey *post hoc* test). Boxplots include the median value (dark line) and the whiskers represent a 1.5 interquartile range. **(D)** Behavioral data from DanioVision motion tracking shows significant increased distance moved over the entire 1 h for het versus WT siblings (^∗^*p* < 0.05 using a Dunn’s multiple comparison *post hoc* test). The left plot indicates the mean distance per minute with error bars representing standard error of the mean. Indicated by the arrow, at 45 min fish were subjected to light flashing (see Materials and Methods). The box plot shows the mean for the 1 h behavioral assay, the median value (dark line) and the whiskers represent a 1.5 interquartile range.

Though the platform itself impacts morphometric and behavioral features in WT larvae, we hypothesized that the effects would be equal across all fish, thus making it possible to parse genotype effects on development and behavior in mutant larvae. To test this, we used CRISPR to generate a null mutant zebrafish model of human *SYNGAP1*, a gene encoding neuronal Ras and Rap GTPase-activating proteins. Heterozygous (het) mutations of *SYNGAP1*, important for synaptogenesis and regulation of excitatory synapses (Rumbaugh et al., 2006; Walkup et al., 2015), are associated with neurodevelopmental disorders, including epilepsy, ASD, and ID (Gamache et al., 2020). A previous study using morpholinos targeting mRNAs encoding the two zebrafish orthologs of the gene (Supplementary Figure S5A) *syngap1a* and *syngap1b* showed malformed mid- and hindbrain [48 h post fertilization (hpf)], developmental delay (48 hpf), and spontaneous seizures, correlated to an observed decrease in GABA neurons in the mid- and hindbrain, specific to the *syngap1b* morphant larvae at 3 dpf (Kozol et al., 2015). Although functional domains from *syngap1a* and *syngap1b* are conserved in zebrafish, the characterization of *syngap1a* was not performed in the aforementioned study due to low penetrance of the morpholino and toxicity of using higher doses (Kozol et al., 2015). The longitudinal expression profile of *syngap1b* is relatively low until 30 hpf based on previously published RNA-seq data (White et al., 2017; Supplementary Figure S5B), suggesting that morpholinos, which are best suited for knocking down early developmental genes, may not represent an optimal approach to characterize *syngap1b*. As such, we generated a stable mutant line *syngap1b^tup1^* carrying a 23-bp frame-shifting deletion predicted to produce a transcript encoding a 213-amino acid protein (84.12% shorter than the full-length protein) (Figure 4A). Larvae carrying the deletion exhibit reduced expression of *syngap1b* in a dosage-dependent fashion, with hom mutants quantified as having the least amount of transcript (Supplementary Figure S5C). This suggests that mutant transcripts undergo nonsense-mediated decay (NMD) resulting in a loss of gene function. To ensure that any phenotype produced in *syngap1b^tup1^* was not a product of off-target mutations, we also assayed the top 13 genetic loci predicted by CIRCLE-seq (Tsai et al., 2017) to be edited and detected no indels (Supplementary Table S1 and Supplementary Figure S5D), including in the *syngap1a* paralog.

We collected data on siblings (*n* = 124) produced by crossing *syngap1b^tup1/+^* lines, creating a mix of WT, het, and homozygous (hom) larvae, using our multiphenotyping platform (Supplementary Table S4). We observed a skew in genotype frequency (*n* = 48 het, *n* = 33 hom, and *n* = 43 WT; *p* = 0.02 χ^2^ test) from the expected Mendelian distribution suggesting that *syngap1b^tup1^* mutants (and particularly het larvae) may be less viable compared to their WT siblings. We did not observe an enrichment of larvae carrying mutant alleles falling outside of a normal distribution of morphometric measurements, ruling out highly penetrant developmental defects associated with null mutations of *syngap1b* (Supplementary Figure S5E). Considering all morphometric data in combination, we performed a PCA and identified that L, EA, OT, and Tel all contributed significantly to the dispersion of our data (all squared loadings >0.60 for first dimension) (Supplementary Table S5 and Figure 4B). Therefore, we analyzed our morphometric data using models that included each measurement as a covariate to identify individual effects of *syngap1b* genotype. This combined approach revealed larger EA (4.6% increase, *p* = 4.16 × 10^–5^ Tukey’s *post hoc*), OT (3.1% increase, *p* = 2.7 × 10^–6^ Tukey’s *post hoc*), and Tel (4.1% increase, Tukey *post hoc*: *p* = 0.016) in hom *syngap1b^tup1/tup1^* larvae at 3 dpf relative to their WT siblings (Supplementary Table S5 and Figure 4C). EA was also suggestive to be larger on hom *syngap1b^tup1/tup1^* at 5 dpf (*p* = 0.053 Tukey *post hoc*). Notably, hom *syngap1b^tup1/tup1^* larvae did not exhibit increased L (3 dpf: *p* = 0.267; 5 dpf: *p* = 0.348 Tukey *post hoc*) suggesting that mutant larvae were not generally larger overall. Next, we assessed seizure susceptibility of larvae when treated with PTZ and found a significant increase in movement for het *syngap1b^tup1/+^*^–^mutants (mean distance = 177.38 mm, *p* = 0.047 Dunn’s multiple comparison *post hoc*) compared with their WT siblings (mean distance = 138.50 mm) (Supplementary Table S6 and Figure 4D). Surprisingly, despite the developmental defects observed in *syngap1b^tup1/tup1^* mutants, no significant behavioral difference was observed for hom mutants (mean distance = 150.56 mm) versus WT siblings. We note that from 10 to 45 min hom mutants appear to exhibit greater movement compared to WT suggesting altered seizure behavior, although different from het mutant siblings, which exhibit greater movement across these same timepoints. Comparing genotype effects in larvae not treated with PTZ resulted in no significance overall (Supplementary Figure S5F).

### A Novel Zebrafish Model for ASD-Candidate Gene *SLC7A5*

**FIGURE 5:**
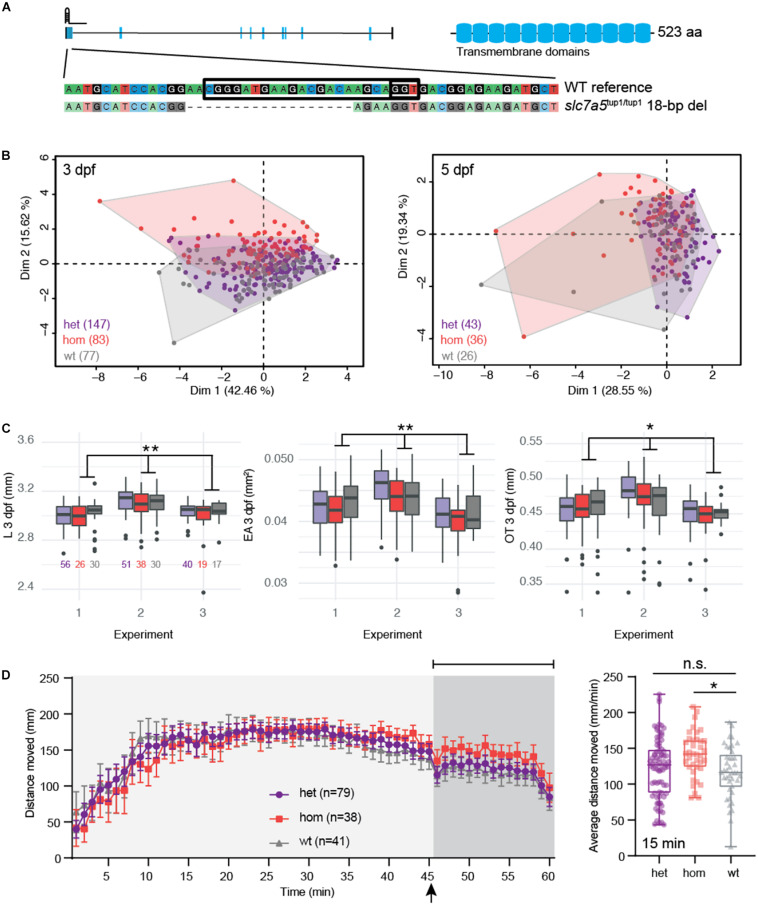
*slc7a5* CRISPR hom mutants exhibit moderate developmental delay and enhanced seizures with flashing lights. **(A)** The zebrafish mutant line *slc7a5*^tup1/tup1^ was generated using a single gRNA targeting the first exon of the gene resulting in an 18-bp deletion leading to a frameshift. **(B)** PCA of combined morphometric traits at 3 and 5 dpf colored by genotype. **(C)** The three traits found to be significantly increased in het (purple) and hom (red) mutant versus WT (gray) siblings included L, EA, and OT at 3 dpf, displayed as box plots with total numbers of larvae measured indicated in the EA plot (^∗^*p* < 0.05; ^∗∗^*p* < 0.01 using a Tukey *post hoc* test). Boxplots include the median value (dark line), and the whiskers represent a 1.5 interquartile range. **(D)** Behavioral data from DanioVision motion tracking shows significant increased distance moved over the final 15 min following flashing lights for hom versus WT siblings (^∗^*p* < 0.05 using a Dunn’s multiple comparison *post hoc* test). The left plot indicates the mean distance per minute with error bars represented as standard error of the mean. Indicated by the arrow, at 45 min fish were subjected to light flashing (see Materials and Methods). The box plot shows the mean for the final 15 min of the behavioral assay, and the median value (dark line) and the whiskers represent a 1.5 interquartile range.

Recently, recessive mutations of *SLC7A5* were reported in children with ASD, microcephaly, and seizures (Tǎrlungeanu et al., 2016). This gene encodes large neutral-amino-acid transporter 1 (LAT1), an essential channel present at the blood–brain barrier in epithelial cells, that facilitates the movement of branched-chain amino acids and other large neutral amino acids from the periphery into the brain (Napolitano et al., 2015). “Knockout” mouse models recapitulated several phenotypic features observed in patients, including ASD-related behaviors, motor coordination abnormalities, and alterations in inhibitory and excitatory neuronal communication. We tested if the knockout of the single zebrafish ortholog *slc7a5* resulted in similar neurodevelopmental defects (Supplementary Figure S6A). Using published RNA-seq whole-embryo data (White et al., 2017), we found that *slc7a5* is expressed starting between 6 and 24 hpf followed by a steady increase throughout larval development (Supplementary Figure S6B). To model a complete hom recessive knockout, we generated the stable mutant line *slc7a5^tup1^* carrying an 18-bp deletion predicted to encode a truncated protein of 17 amino acids (96.75% shorter than the full-length protein) (Figure 5A). Using qRT-PCR, we quantified reduced expression in larvae carrying the deletion, with hom mutants exhibiting the lowest transcript abundance (Supplementary Figure S6C). Like our *syngap1b* mutant, this suggests that transcripts carrying the frame-shift variant are subject to NMD and result in loss of gene function. We verified that no insertions or deletions (indels) existed at the top six off-target sites impacting genes as predicted by CIRCLE-seq that might contribute to phenotypes (Supplementary Table S1 and Supplementary Figure S6D).

Using data collected from siblings (*n* = 337) produced from het G_1_ crosses, we did not observe any significant mortality nor unexpected skew in genotypes (*n* = 161 het, *n* = 90 hom, and *n* = 86 WT; *p* = 0.81 χ^2^ test) showing that *slc7a5^tup1^* mutants are viable compared to their WT siblings (Supplementary Table S7). Examining the distribution of morphometric data, we also did not note any enrichment of mutants in fish exhibiting outlier traits (Supplementary Figure S6E). When considering all features in combination, we noted that hom *slc7a5^tup1/tup1^* mutants clustered separately from the other two genotypes at 3 dpf (Figure 5B). The primary traits driving this deviation were EA, OT, and L (all squared loadings >0.80 for first dimension). In models that accounted for these multiple variables simultaneously, we observed that hom *slc7a5^tup1/tup1^* mutants exhibited significant, although modest, differences in EA (0.9% decrease, *p* = 7.5 × 10^–3^ Tukey *post hoc*), OT (0.1% decrease, *p* = 0.023 Tukey *post hoc*), and L (0.6% decrease, *p* = 9.9 × 10^–3^ Tukey *post hoc*) at 3 dpf (Supplementary Table S5 and Figure 5C). The overall reduction of EA, OT, and L suggests that larvae may experience minor developmental delay. At 5 dpf, no measurement showed differences between *slc7a5* genotypes. Furthermore, we quantified significantly greater movement in PTZ-treated larvae following light flashes in the final 15 min, suggesting enhanced seizures in hom *slc7a5^tup1/tup1^* mutants (mean distance = 141.3 mm, *p* = 0.013 Dunn’s multiple comparison *post hoc*) versus WT (mean distance = 116.7 mm) that was not detected for *slc7a5^tup1/+^* het mutant siblings (mean distance = 122.8 mm; Supplementary Table S6, Figure 5D). This observation is in line with the recessive nature of *SLC7A*5 mutations associated with defects identified in children with ASD. Notably, we did not identify any significant differences across genotypes when considering distance moved over the entire 1 h nor for larvae not subjected to PTZ (Supplementary Figure S6F).

To test if our combinatorial phenotyping approach was feasible using a CRISPR G_0_ mosaic mutant model, we compared effects on embryos injected simultaneously with three gRNAs targeting *slc7a5* (*n* = 101; “pool” mutant) with “mock” controls (*n* = 72) (Supplementary Table S8). We verified significant indel generation at each individual target site by assessing the smear produced from PCR amplicons of target sites from injected larvae ran on a polyacrylamide gel (data not shown). From this, we observed the same cluster separation between mutants and controls (Supplementary Figure S7A) as we did between WT and hom mutants (Figure 5B) at 3 dpf. We also recapitulated the reduction in L (2.4%, *p* = 2.5 × 10^–4^ Tukey *post hoc*), but alternatively observed an increase in OT (1.3%, *p* = 0.05 Tukey *post hoc*), which was further verified by an increase in Tel (1.6%, *p* = 0.01 Tukey *post hoc*) at 3 dpf (Supplementary Table S5 and Supplementary Figure S7B). Finally, we observed reduced movement of PTZ-treated pool mutants (mean distance = 138.4 mm) versus mock controls (mean distance = 164.8 mm) across 1 h of motion tracking using the behavioral assay (Supplementary Figure S7C), counter to results in the *slc7a5^tup1^* stable mutants (Figure 5C), although the comparisons of the overall averages is not significant. Examination of motion-tracking video shows larvae exhibiting what appear to be stage III seizures (Baraban et al., 2005), potentially explaining the reduction in movement and suggesting a more severe phenotype may be observed in mosaic versus stable mutant lines. Less severe phenotypes have been reported in CRISPR stable mutants that produce transcripts subject to NMD compared with zebrafish subjected to morpholinos or mosaic mutations of the same gene due to compensation (El-Brolosy and Stainier, 2017; El-Brolosy et al., 2019).

## Discussion

Several studies have proposed morphological and behavioral phenotyping platforms for the characterization of zebrafish larvae (Pardo-Martin et al., 2010; Reif et al., 2016; Liu et al., 2017; Teixidó et al., 2019). However, previous literature has not assessed the impact of combining automated VAST morphometric and DanioVision behavioral assays in the same larvae and particularly for the purpose of testing CRISPR mutants. Here, our goal was to determine if imaging with VAST or motion tracking would alter zebrafish behavior or developmental features, in addition to optimizing parameters to maximize the number of measurements obtained for each larva. Contrary to a previous study (Pardo-Martin et al., 2010), we found that imaging 3 dpf larvae using VAST significantly impacted downstream behavior and development measured at 5 dpf. Possible contributing factors include the temporary anesthesia and physical transport through capillaries necessary for imaging larvae with VAST. We did not directly test the individual effects of these variables in our study, though Pardo-Martin et al. (2010) show they do not significantly impact downstream phenotypes in larvae imaged at 2 dpf. Further, we note the low MS-222 concentration used in our studies (76 μM or 20 mg/L) (Chang et al., 2012) would suggest that impacts on development might be minimal. Nevertheless, previous studies have shown that focal exposure to varying concentrations of MS-222 (191–574 μM) at earlier developmental time points (before 26 hpf) leads to cartilage malformations (Félix et al., 2018) and upregulation of genes associated with apoptosis (Félix et al., 2020). There is a considerable body of literature demonstrating the negative effects of anesthesia in the developing human brain (Andropoulos, 2018), with most studies finding associations with neuronal apoptosis specifically when using anesthetics. More relevant to our study is the cell death by necrosis and apoptosis observed when using local anesthetics, which act in voltage gated sodium channels, as is the case with MS-222 (Ferreira et al., 2018). However, the effects of MS-222, an anesthetic used in fish not humans, have not been well studied in the developing nervous system of zebrafish (Topic Popovic et al., 2012), and future studies are needed. Overall, despite the impacts on developmental features, the benefits of using the VAST platform are significant, as it facilitates automated measurements in living larvae thus preventing manual errors, improving consistency, and increasing speed of measurements.

To assess whether our platform was capable of identifying defects in spite of impacts on measured features, we proceeded with characterizing developmental and behavioral phenotypes of two novel CRISPR-generated mutant zebrafish lines of ASD-associated genes (*SYNGAP1* and *SLC7A5*). We assayed a stable mutant line of *syngap1b* as a proof of concept, a gene that had previously been studied using morpholinos in zebrafish embryos (Kozol et al., 2015). We noted a skewed distribution of genotypes (reduced hom and het alleles versus WT) after subjecting them to our combined phenotyping assay, suggesting that mutant larvae have reduced viability, in line with perinatal lethality of hom null mutations observed in *Syngap1* mouse models (Gamache et al., 2020). Our het *syngap1b^tup1/+^* mutants moved greater distances over 1 h in the presence of PTZ versus WT, suggesting they may be experiencing enhanced seizures, consistent with spontaneous seizures in *syngap1b* morphants (Kozol et al., 2015). In contrast, hom *syngap1b^tup1/tup1^* mutants did not significantly differ from WT or hom mutants in their overall movement, suggesting they perhaps exhibit intermediate seizure effects (Figure 4D). Notably, our assay does not currently score for spontaneous clonic seizures observed previously in morphants but may explain the reduction in movement of our hom versus het mutants; as such, automated methods to detect such behaviors in PTZ-treated and -untreated larvae from motion-tracking videos represents a future direction. Furthermore, our morphometric data suggests that *syngap1b^tup1^* het and hom mutants exhibit significantly increased head and eye sizes at 3 dpf, counter to the findings in zebrafish morphants (Kozol et al., 2015), mutant mice (Kilinc et al., 2018), and patients (Gamache et al., 2020) that exhibit reduced brain ventricle sizes and mild microcephaly. It is important to note that microcephaly phenotypes have low penetrance in *SYNGAP1* zebrafish morphants and are identified in 30% of human patients carrying heterozygous mutations of the gene (Parker et al., 2015). Traits with low penetrance are difficult to detect using our higher-throughput pipeline, which compares means across groups requiring a larger number of fish to detect sporadic differences.

Finally, we generated a novel stable mutant of *slc7a5*, a gene never before assayed in zebrafish, with the goal of recapitulating microcephaly and seizure phenotypes observed in human patients and mouse models carrying hom recessive mutations (Tǎrlungeanu et al., 2016). Our PCA analysis of all morphometric features at 3 dpf showed that hom *slc7a5^tup1/tup1^* mutants clustered separately from WT and het siblings (also observed between our pool mutants and mock controls in the mosaic G_0_ assay), though individual traits were not significantly different between genotypes. Since our assay quantified multiple traits from the same larvae, we could parse individual effects of genotype on each measurement by considering each measured trait as a covariate. By doing this, we detected small yet significant reductions in L, EA, and head width (OT) in our hom *slc7a5^tup1/tup1^* mutants at 3 dpf compared to both het and WT. Notably, in mosaic G_0_ 3 dpf mutant larvae, we recapitulated decreased L with larger effect but observed increased head width (OT and Tel), counter to findings in the stable *slc7a5^tup1^* mutant line and human patients. Though unclear, it is possible that discordant results could be due to off-target mutations produced from injecting three gRNAs in our pool mutants. Furthermore, hom larvae exhibited increased movement, which could indicate enhanced seizures, in the presence of light flashing but not over the entire 1 h when treated with PTZ. Although not reported in the few *SLC7A5* patients published to date, this suggests that patients may be susceptible to light-flashing-induced seizures, as is the case with certain types of epilepsy in humans (Galizia et al., 2015). For both *slc7a5* and *syngap1b*, we did not observe any strong mutational effects in non-PTZ treated larvae. For future studies, we will consider incorporating additional perturbations during motion tracking with known associations to enhanced behaviors and seizure phenotypes, such as heat (Warner et al., 2017; Peron et al., 2018) and startle (Tegelenbosch et al., 2012).

In all, we have demonstrated that our multi-phenotyping platform can detect subtle morphological and behavioral changes in two ASD mutant zebrafish models. This approach, coupled with CRISPR mutant screens of many genes in parallel, either in G_0_ mosaic or stable mutant lines, represents a powerful first-pass to quickly identify genes impacting development and behavior. Though we focus our study here on ASD genes, most of the traits we assessed are not unique to neurodevelopment; thus, additional studies are required to pinpoint affected brain features. Future work includes expanding our phenotypes to include more neurological traits. In addition to the aforementioned improvements to our assay to induce and detect spontaneous seizures, we aim to extract more phenotypes from our VAST images, in addition to L, EA, Tel, and OT. By incorporating transgenic reporter fish, such as those with labeled excitatory and inhibitory neurons to identify neuronal transmission imbalance or calcium reporters such as GCaMP to further characterize seizures, coupled with a higher resolution fluorescent microscope and VAST imaging, our assay could more comprehensively characterize specific brain defects in our disease mutant models. Furthermore, a current limitation to measuring additional traits is the reliability of existing automated methods to accurately and consistently extract features. Although many of our measurements were performed automatically with the FishInspector software, we manually inspected every image due to issues with feature tracing to ensure accuracy and, in some cases, deferred to manual measurements (OT and Tel) or removed features completely from our analyses (head width, yolk area, and pericardium area). Future goals include improvements in automated extraction of features as well as integrating data from multiple sources to pinpoint genotype effects.

## Data Availability Statement

The data has been deposited to the European Nucleotide Archive Accession PRJEB40101.

## Ethics Statement

The animal study was reviewed and approved by the University of California Davis Institutional Animal Care and Use Committee.

## Author Contributions

AC-R and MD designed the experiments, administered, and supervised the project. AC-R, JU-S, and MD contributed to the conceptualization and evolution of the research. JU-S generated data analysis and visualization software. AC-R, JU-S, KW, AS, AQ, GK, EJ, and BR performed the experiments. AC-R, JU-S, AS, AQ, KW, EJ, and MD analyzed the data. AC-R, JU-S, KW, and MD wrote the original manuscript. BR and PL provided feedback and edits for the manuscript. MD acquired the financial support for this project. MD and PL provided resources for this project. All authors approved the submitted version of the article.

## Conflict of Interest

The authors declare that the research was conducted in the absence of any commercial or financial relationships that could be construed as a potential conflict of interest.
