# Supplementary material for: Assessment of Autism Zebrafish Mutant Models Using a High-Throughput Larval Phenotyping Platform
Source: Front Cell Dev Biol. 2020 Nov 23;8:586296. doi: 10.3389/fcell.2020.586296 (PMC7719691; doi:10.3389/fcell.2020.586296)
Supplement: Supplementary file 1 [file Data_Sheet_1.pdf]

SUPPLEMENTARY MATERIAL

Assessment of autism zebrafish mutant models using a high-throughput larval phenotyping platform

Alexandra Colón-Rodríguez[1], José M. Uribe-Salazar[1,2], KaeChandra B. Weyenberg[1], Aditya Sriram[1], Alejandra Quezada[1,3], Gulhan Kaya[1], Brittany Radke[1], Emily Jao[1], Pamela J. Lein[4,5], Megan Y. Dennis[1,2,5]

[1]Genome Center and Department of Biochemistry & Molecular Medicine, School of Medicine, University of California, Davis, CA, USA; [2]Integrative Genetics and Genomics Graduate Group, University of California, Davis, CA, USA; [3]Sacramento State RISE Program, California State University, Sacramento, CA, USA; [4]Department of Molecular Biosciences, School of Veterinary Medicine, University of California, Davis, CA, USA; [5]MIND Institute, School of Medicine, University of California, Davis, CA, USA

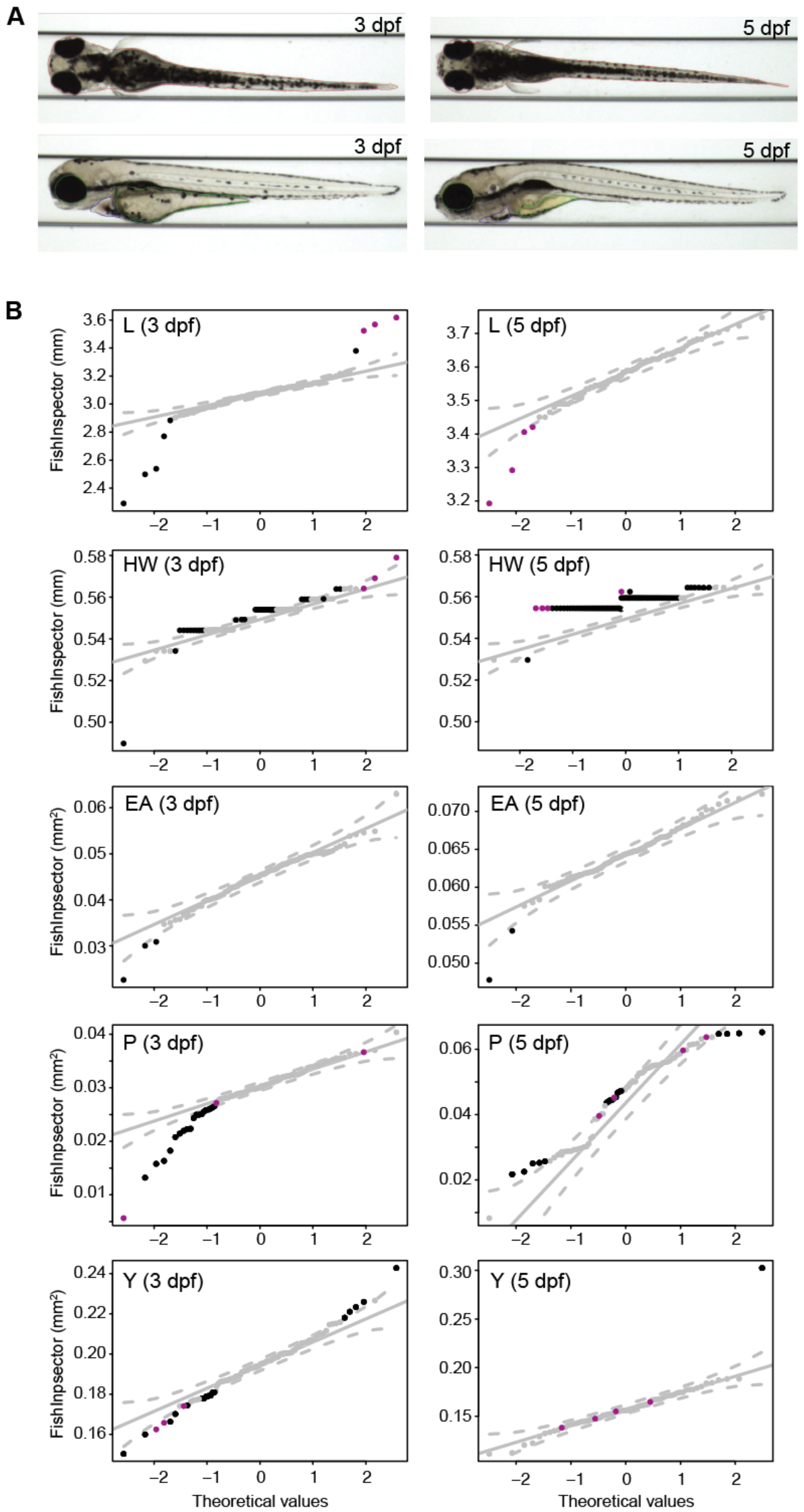

Figure S1. Automated FishInspector quantification of morphometric features. (A) Examples of zebrafish larvae at 3 and 5 dpf in dorsal and lateral view with FishInspector features highlighted. **(B)** Q-Q plots of each morphometric measurement (L, length, HW, head width; EA, average eye area; P, pericardium; Y, yolk) extracted from FishInspector with outliers in black with individuals highlighted purple if technical issues in imaging were observed.

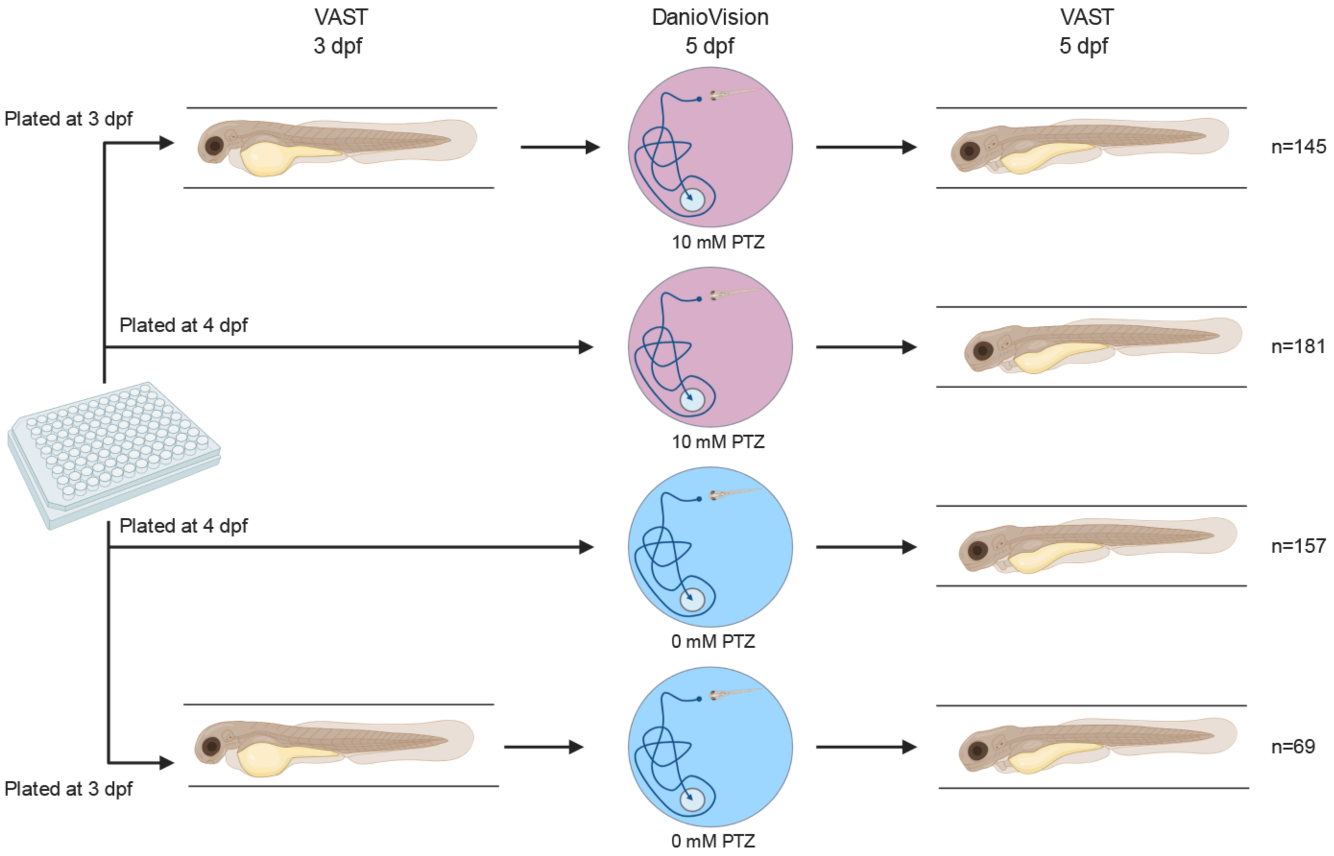

Figure S2. Combined morphometric and behavioral experimental conditions tested. Wild-type NHGRI-1 larvae were subjected to different combinations of morphometric and behavioral tests across seven different experiments to determine their impacts on final phenotypic measurements. Not pictured are larvae that were tested with with a single experimental condition, including: behavioral measurements using the DanioVision at 5 dpf with 10 mM PTZ (n=52) and 0 mM PTZ (n=142) as well as morphometric measurements using the VAST at 3 dpf (n=185) and 5 dpf (n=61).

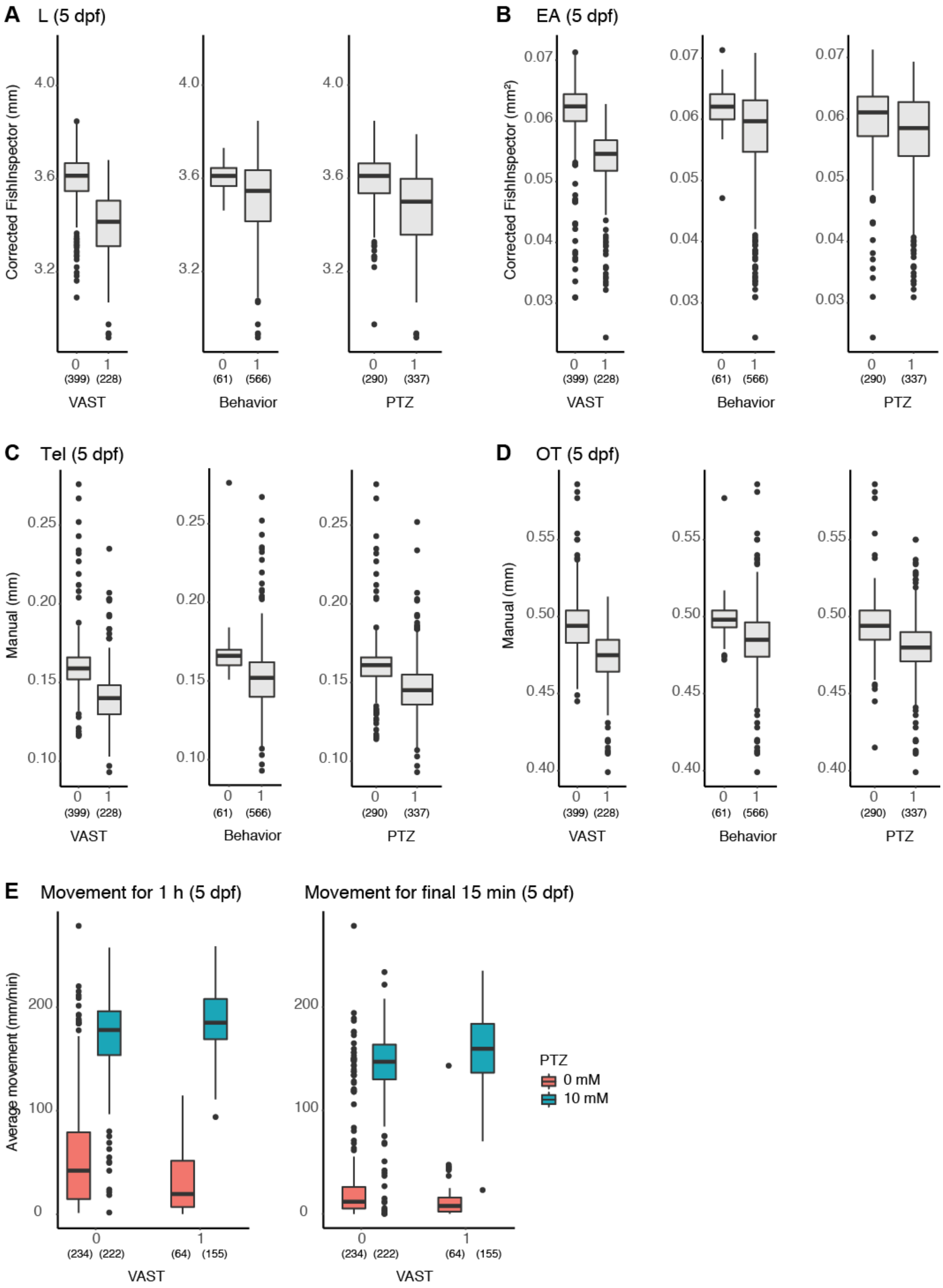

Figure S3. Impact of morphometric and behavioral measurements by treatment variables. Morphometric measurements of 5 dpf larvae subjected to varied treatments (VAST at 3 dpf, behavior at 5 dpf, and 10 mM of PTZ denoted with 0=no and 1=yes) as determined by the VAST system quantified via corrected FishInspector mappings for **(A)** L and **(B)** EA and via manual measurements for **(C)** Tel and **(D)** OT are shown as boxplots. **(E)** Average movement per minute quantified for the entire 1 h or the final 15 min after light flashing is shown as boxplots for larvae subjected to VAST measurements at 3 dpf (0=no and 1=yes) and at varied PTZ concentrations (red=0 mM and blue=10 mM). Total numbers of measured larvae are indicated in parentheses.

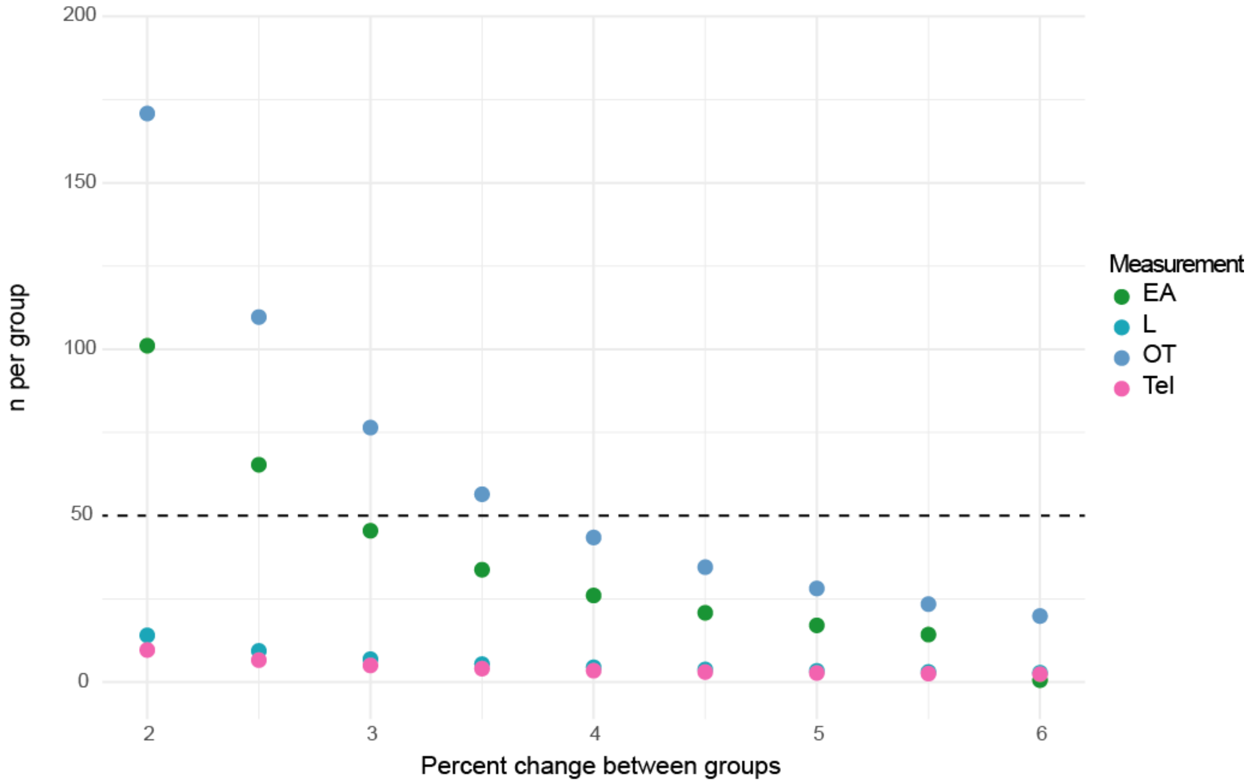

Figure S4. Power to detect phenotypic differences from morphometric measurements using the combined platform. For the four morphometric features measured at 5 dpf, we calculated how many samples are needed per group to achieve 80% power to detect varied percentages of changes between tested groups. For all traits, testing 50 larvae per group allows 4% change between measurements to be detected (shown by the dashed line).

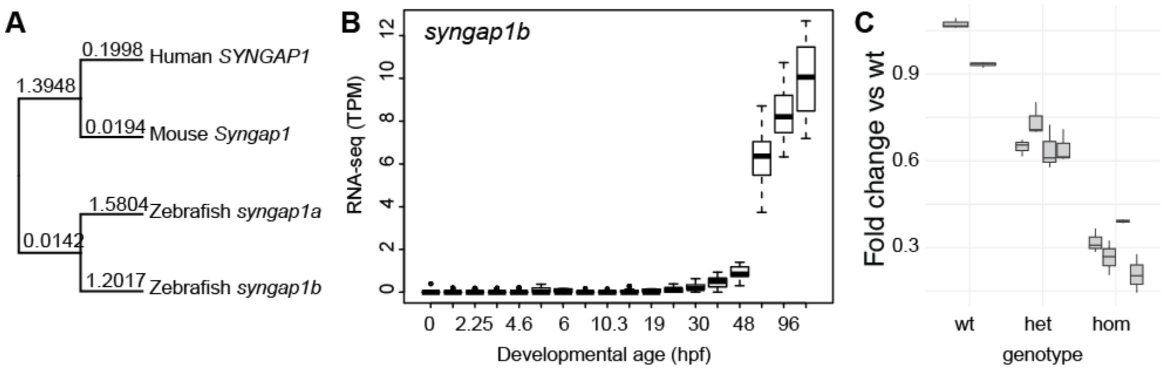

D

Target	Position	RPMR	Description	Sequence
ON	chr16:45,603,573-45,603,596	132.8	*syngap1b* exon 5	AGCGAGGGAGGATCATGTGGCGG
OFF1	chr18:33,539,669-33,539,692	496.2	>10 Kb from gene	••TT••••••A•C••••••AT••
OFF2	chr2:58,886,754-58,886,777	379.3	*mau2* intron 17	•••••••••A•••••CA•••A••
OFF3	chr19:258,396-258,419	366.2	*syngap1a* exon 5	•T••T•••••A••••••••••••
OFF4	chr16:7,265,679-7,265,702	292.3	CU466278.2 intron 1	••T••••••AAC•••••••AA••
OFF5	chr10:17,857,061-17,857,084	284.5	*phf24* intron 1	••G•••••••••AT•GA•••A••

RPMR: reads per million reads mapped [Reads / Total mapped reads * 1000000]
Total reads mapped = 7,198,150
• = same base

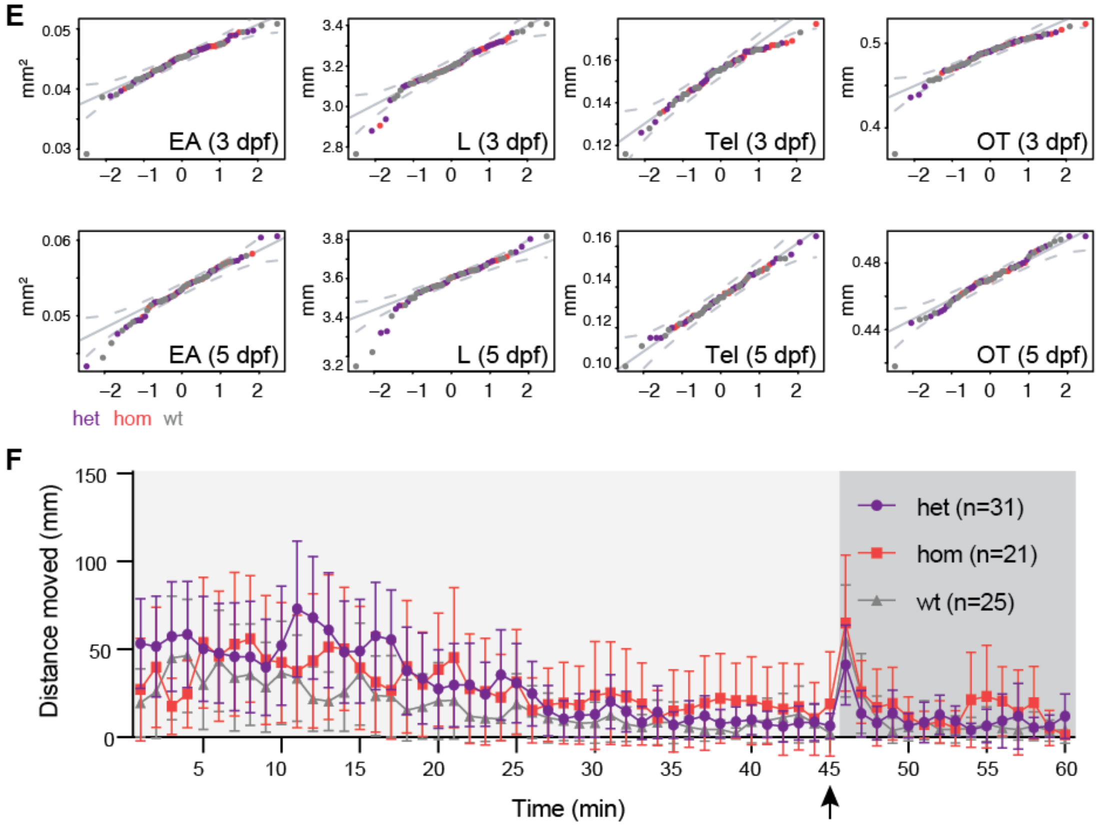

Figure S5. Generation and characterization of *syngap1b* CRISPR mutant. (A) Phylogram indicates that two paralogs exist in zebrafish, *syngap1a* and *syngap1b*. Branch lengths were determined using a multiple-sequence alignment of CDS using the maximum-likelihood approach in MEGA7. **(B)** Longitudinal *syngap1b* expression in whole zebrafish embryos is plotted as transcript per million (TPM) using previously published data from White *et al.* (2017). **(C)** qRT-PCR of RNA extracted from 5 dpf *syngap1b*^{tup1} siblings carrying the deletion leads to reduced *syngap1b* expression suggesting loss of gene function. Each box plot represents three technical replicates of an individual larvae plotted as fold change to WT average normalized by β-actin. **(D)** Top off-target sites hitting genes were identified for the gRNA used to generate the *syngap1b*^{tup1} mutant using CIRCLE-seq. **(E)** Q-Q plots of each morphometric measurement with genotypes colored. Plotted on the x-axes are theoretical values from a normal distribution. **(F)** Behavioral data from DanioVision motion tracking over 1 h without PTZ treatment, with genotypes colored as indicated. The arrow represents when flashing lights were administered for 1 min after 45 min of tracking. The plot indicates the mean distance per minute with error bars represented as standard error of the mean.

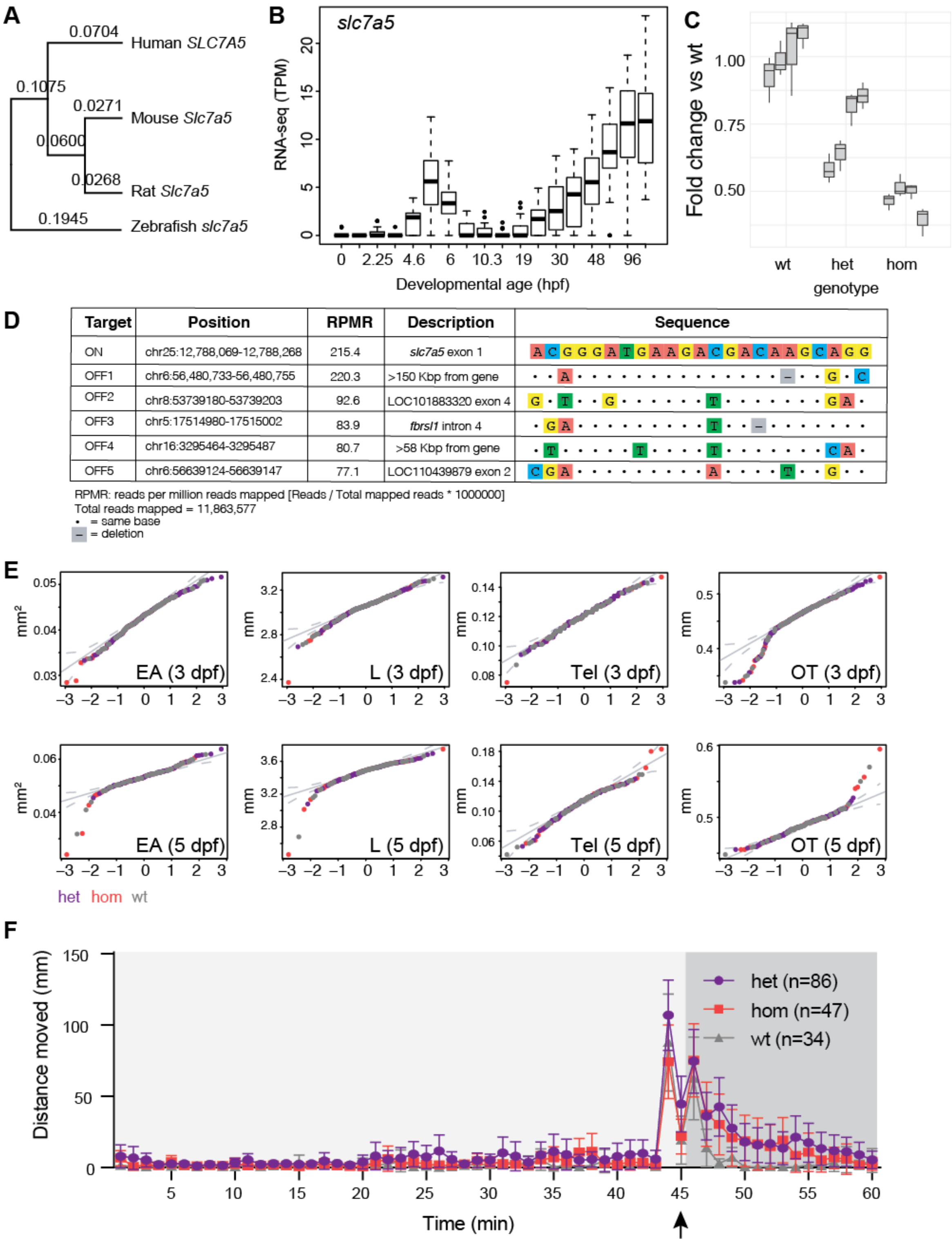

Figure S6. Generation and characterization of *slc7a5* CRISPR mutant. (A) Phylogram indicates that one ortholog exists in zebrafish, *slc7a5*. Branch lengths were determined using a multiple-sequence alignment of CDS using the maximum-likelihood approach in MEGA7. **(B)** Longitudinal *slc7a5* expression in whole zebrafish embryos is plotted as transcript per million (TPM) using previously published data from White *et al.* (2017). **(C)** qRT-PCR of RNA extracted from 5 dpf *slc7a5*^{tup1} siblings carrying mutations leads to reduced *slc7a5* expression suggesting loss of gene function. Each box plot represents three technical replicates of an individual larvae plotted as fold change to WT average normalized by β-actin. **(D)** Top off-target sites hitting genes were identified for the gRNA used to generate the *slc7a5*^{tup1} mutant using CIRCLE-seq. **(E)** Q-Q plots of each morphometric measurement with genotypes colored. Plotted on the x-axes are theoretical values from a normal distribution. **(F)** Behavioral data from DanioVision motion tracking over 1 h without PTZ treatment, with genotypes colored as indicated. The arrow represents when flashing lights were administered for 1 min after 45 min of tracking. The plot indicates the mean distance per minute with error bars represented as standard error of the mean.

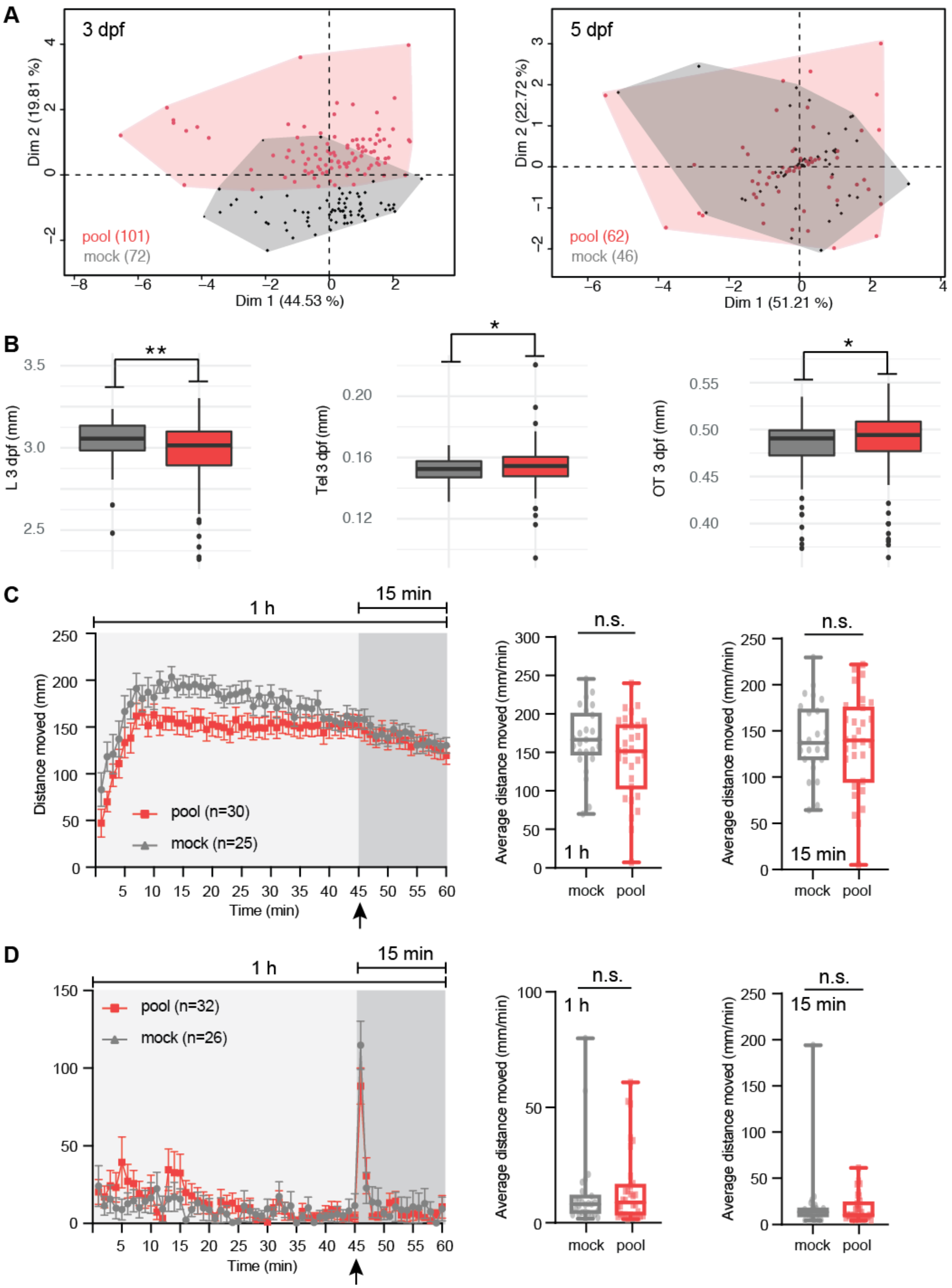

Figure S7. ***slc7a5*** **CRISPR 'pool' mosaic mutants exhibit minor developmental defects and reduced activity indicative of clonic seizures in presence of PTZ. (A)** Comparing CRISPR pool mutants versus 'mock' contols, PCA plots of combined morphometric traits at 3 and 5 dpf colored by genotype are shown. **(B)** The three traits found to be significantly altered in mutants versus controls included L, OT, and Tel at 3 dpf, displayed as box plots with total numbers of larvae measured indicated in the PCA plot (*, $p < 0.05$; **, $p < 0.01$ using a Tukey post-hoc test). Boxplots include the median value (dark line) and the whiskers represent a 1.5 interquartile range. Behavioral data from DanioVision motion tracking, represented in plots for larvae treated with **(C)** PTZ and **(D)** without PTZ. For PTZ-treated fish, a decrease in average distance moved is observed in the first 45 minutes in mutant versus mock controls, though this result is not statistically significant (n.s. using a Mann-Whitney test). The left plots indicate the mean distance per minute with error bars represented as standard error of the mean. The box plots show the mean for the entire hour and final 15 min of the behavioral assay, the median value (dark line) and the whiskers represent a 1.5 interquartile range.
